# Effect of Treatment with Cyanidin-3-O-**β**-D-Glucoside on Rat Ischemic/Reperfusion Brain Damage

**DOI:** 10.1155/2012/285750

**Published:** 2012-09-13

**Authors:** Claudia Di Giacomo, Rosaria Acquaviva, Rosa Santangelo, Valeria Sorrenti, Luca Vanella, Giovanni Li Volti, Nicolantonio D'Orazio, Angelo Vanella, Fabio Galvano

**Affiliations:** ^1^Section of Biochemistry, Department of Drug Science, University of Catania, 95125 Catania, Italy; ^2^Department of Pediatric Cardiology and Heart Surgery, IRCCS Policlinico San Donato, 20097 San Donato Milanese, Italy; ^3^Department of Biomedical Science, Human Nutrition Unit, University of Chieti, 66013 Chieti, Italy

## Abstract

This study investigated the effect of cyanidin-3-O-**β**-glucoside on an experimental model of partial/transient cerebral ischemia in the rats in order to verify the effectiveness of both pre- and posttreatments. Cyanidin-3-O-**β**-glucoside-pretreated rats were injected with 10 mg/Kg i.p. 1 h before the induction of cerebral ischemia; in posttreated rats, the same dosage was injected during reperfusion (30 min after restoring blood flow). Cerebral ischemia was induced by bilateral clamping of common carotid arteries for 20 min. Ischemic rats were sacrificed immediately after 20 min ischemia; postischemic reperfused animals were sacrificed after 3 or 24 h of restoring blood flow. Results showed that treatment with cyanidin increased the levels of nonproteic thiol groups after 24 h of postischemic reperfusion, significantly reduced the lipid hydroperoxides, and increased the expression of heme oxygenase and **γ**-glutamyl cysteine synthase; a significant reduction in the expression of neuronal and inducible nitric oxide synthases and the equally significant increase in the endothelial isoform were observed. Significant modifications were also detected in enzymes involved in metabolism of endogenous inhibitors of nitric oxide. Most of the effects were observed with both pre- and posttreatments with cyanidin-3-O-**β**-glucoside suggesting a role of anthocyanin in both prevention and treatment of postischemic reperfusion brain damage.

## 1. Introduction

Cerebral ischemia is defined as a condition where the brain or its parts do not receive enough blood flow to maintain normal neurological function. This causes metabolic changes and possibly cellular death. Restoration of blood flow, although necessary for brain survival, could lead to excessive reactive oxygen species (ROS) formation and nitric oxide synthase (NOS) activation with resulting oxidative/nitrosative stress. Thus, cerebral ischemia and reperfusion can produce neuronal damage triggering a complex series of biochemical events that affect structure and function of brain. 

Mechanisms implicated in neuronal death are complex and multifactorial, also involving intracellular Ca^++^ overload with consequent activation of nitric oxide synthase (NOS), the enzyme that catalyses the synthesis of nitric oxide (NO), a small reactive gas acting both as a signalling molecule and a neurotoxin [1–4]. Several experimental evidence demonstrated a dual role for NO following an ischemic episode [5–7]; in fact, it can exert detrimental and beneficial effects depending on several factors such as the isoform of NOS involved, the amount of NO released, the time and the site of NO production. To this regard, NO produced by endothelial NOS (eNOS) immediately after ischemic attack, promoting vasodilatation, may play a protective role. Later, when overactivation of neuronal NOS (nNOS) and *de novo* expression of inducible NOS (iNOS) occur, the contribution of NO to brain damage becomes relevant.

An increasing number of reports in the literature indicate that endogenously produced inhibitors of NOS, particularly asymmetric dimethylarginine (ADMA), regulate NO generation, and then may be implicated in the pathophysiology of several disorders [8–12]. The major pathways for ADMA elimination are via renal clearance and via metabolism to L-citrulline by the intracellular enzymes dimethylarginine dimethylaminohydrolase (DDAH 1 and DDAH 2) and it is increasingly apparent that metabolism of ADMA is highly regulated [13]. Thus, regulation of DDAHs might represent a novel mechanism by which ADMA levels can be modulated to regulate NO generation and to evaluate possible therapeutical options [14].

Although the pathobiological mechanisms of ischemia/reperfusion injury are multifactorial, oxidative stress seems to represent the common final path [15]. Recently, intense interest has focused on the antioxidant properties of natural products. In particular, natural products may act by preventing the free radical generation, neutralizing free radicals by nonenzymatic mechanisms, and/or by enhancing the activity of endogenous antioxidants [16] such as stress-inducible proteins. 

Heme oxygenase (HO) (EC 1.14.99.3) is a microsomal enzyme that oxidatively cleaves heme and produces biliverdin, carbon monoxide (CO) and iron [17]. To date, two isoforms of HO have been identified: HO-1, or inducible enzyme, and HO-2 or constitutive isoform [17–21]. A substantial body of evidence demonstrates that HO-1 induction represents an essential step in cellular adaptation to stress subsequent to pathological events [13, 22–25]; then HO-1 hyper-expression can be considered both a marker of cellular stress and also regarded as a potential therapeutic target in a variety of oxidant-mediated diseases [26]. 

Recently it has been reported that polyphenolic natural compounds are able to induce potently HO-1 expression, exercising protective effects [27–29]. As a consequence, the beneficial actions attributed to several natural substances could be also due to their intrinsic ability to activate the HO-1 pathway [27–29]. The list of natural compounds acting as antioxidants includes anthocyanins, a widespread group of water-soluble plant constituents collectively known as flavonoids.

Cyanidin-3-O-*β*-D-glucoside (C3G) is a natural compound whose antioxidant, anti-inflammatory, and iron-chelating properties have been demonstrated in numerous studies using several methods, both *in vivo* and *in vitro* [30–33]. 

The present *in vivo* study was performed to verify whether the treatment with C3G is able to counteract oxidative stress induced by postischemic reperfusion and if its effect may be mediated by HO-1. In addition, the possibility of an interference of C3G on DDAH/NOS pathway was also tested.

## 2. Material and Methods

### 2.1. Animals

Male Wistar rats (100–120 g b.w.) were fed a certified balanced diet and kept in temperature (20 + 1°C) and humidity (50%) controlled rooms, caged with raised floors of wide mesh. The animals were deprived of food for 12 hours before experiment but allowed free access to water. All the experimental procedures reported in this study were approved by the Animal Care and Use Committee of University of Catania, Italy (approval number 037, prot. 37394 TIT cc VIII/2). 

### 2.2. Experimental Protocols

For experiments, animals were anaesthetized by ethyl urethane (1.2 g/kg b.w., i.p.); cerebral ischemia was induced by bilateral clamping of common carotid arteries for 20 min. The induction of ischemia was confirmed by measuring lactate levels. A lot of untreated, sham-operated animals was used as control. C3G-pretreated and post-treated sham-operated rats were also included in the experimental protocol. Sham-operated animals did not undergo ischemia and reperfusion: they were anesthetized, their skin was incised, and the carotid arteries were exposed, but not occluded. All the animals were sacrificed by injection of an overdose of anaesthetic. Rats were randomly divided into 3 groups: (a) saline-treated animals, (b) C3G-pretreated rats, and (c) C3G posttreated rats. C3G-pretreated rats were injected with 10 mg/Kg intraperitoneal (i.p.) 1 h before the induction of cerebral ischemia; in C3G post-treated rats the same dosage of C3G was injected during reperfusion (30 min after restoring blood flow). These times were chosen according to data reported in literature about plasma concentrations of C3G after i.p. administration [34]. Ischemic rats were sacrificed immediately after 20 min of bilateral clamping of carotids; animals subjected to postischemic reperfusion were sacrificed after 3 or 24 h restoring blood flow. Since ischemic rats were sacrificed immediately after 20 min ischemia, we could not administer the cyanidin 30 min after restoring blood flow.

### 2.3. Survival Rate

Percentage of survival was determined by keeping 30 animals, submitted to experimental procedure of 20 min partial cerebral ischemia, under observation for 24 hours. A group of saline-treated, ischemic rats were used as a reference. A lot of sham-operated (both saline- and C3G-pre and posttreated) animals were regarded as control group.

All brains were rapidly removedin a cold room, frozen at −80°C and processed for biochemical analysis within 3 days. Brain tissue was homogenized in 9 volumes of the cold proper buffer. Aliquots of homogenate of each sample were used for determining brain levels of lactate, non proteic thiol groups (RSH) and lipid peroxide (LOOH), for the evaluation of heme oxygenase (HO-1) by specific enzyme-linked immunosorbent assay (ELISA) kit, for expression of *γ*-glutamyl cysteine synthase (*γ*-GCS), endothelial, neuronal, and inducible nitric oxide synthetases (eNOS, nNOS, and iNOS, resp.), DDAH-1 and DDAH-2 by western blot (WB), and for determination of DDAH activity. Results of WB were normalized to *β*-actin and expressed as Arbitrary Units (AU). Protein content was determined by Lowry's method [35]. 

### 2.4. Lactate Level Determination

For the determination of the lactate levels the tissue was homogenized in 20 mM glycylglycine buffer, pH 10, containing 70 mM glutamate; the homogenate was deproteinized by 4% HClO_4_ (final concentration); the spectrophotometric assay was performed following NADH formation at *λ* = 340 nm using Noll's method [36].

### 2.5. Nonproteic Thiol Group Determination

Cerebral levels of non proteic thiol groups (RSH) were measured in 200 *μ*L of brain homogenate using a spectrophotometric assay based on the reaction of thiol groups with 2,2-dithio-bis-nitrobenzoic acid (DTNB) at *λ* = 412 nm (*εM* = 13,600) [37]. Results are expressed as nmoles/mg proteins + S.D.

### 2.6. Determination of Lipid Hydroperoxide Levels

The levels of lipid hydroperoxides were evaluated following the oxidation of Fe^+2^ to Fe^+3^ in the presence of xylenol orange at *λ* = 560 nm [38]. The assay mixture contained, in a total volume of 1 mL: 100 *μ*L of brain homogenate, 100 *μ*M xylenol orange, 250 *μ*M ammonium ferrous sulfate, 90% methanol, 4 mM butylated hydroxytoluene, 25 mM H_2_SO_4_. After 30 min incubation at room temperature, the absorbance at *λ* = 560 nm was measured using a U2000 Hitachi spectrophotometer (Tokyo, Japan). Calibration was obtained using hydrogen peroxide (0.2–20 *μ*M). Results are expressed as nmoles/mg proteins + S.D.

### 2.7. Determination of HO-1 by ELISA

For the determination of HO-1 protein content, 100 *μ*L of brain homogenates were assayed by enzyme-linked immunosorbent assay (ELISA) [39] (Stressgen, VIC, Canada), according to manufacture's instructions. Results are expressed as pg/mg protein + S.D. 

### 2.8. Western Blotting

Brain homogenates were collected for western blot analysis and protein levels were visualized by immunoblotting with antibodies against *γ*-GCS, nNOS, eNOS, iNOS, DDAH-1, or DDAH-2. Briefly, aliquots of homogenate containing 50 *μ*g of proteins were separated by sodium dodecyl sulfate/polyacrylamide gel electrophoresis and transferred to a nitrocellulose membrane. In order to block nonspecific binding sites, the membranes were incubated overnight with 5% nonfat dry milk in 10 mM Tris-HCl (pH 7.4), 150 mM NaCl, 0.05% Tween 20 (TBST) buffer at 4°C. After washing with TBST, the membranes were incubated with a 1 : 1000 dilution of anti-DDAH-1, DDAH-2, or anti *γ*-GCS antibodies, and with 1 : 500 dilution of anti-eNOS, iNOS or n-NOS for 12 hours at 4°C with constant shaking. The filters were then washed and subsequently probed with horseradish peroxidase-conjugated anti-rabbit for *γ*-GCS, eNOS, nNOS, and iNOS at a dilution of 1 : 20000, anti-goat for DDAH-1, and DDAH-2 at a dilution of 1 : 10000. Detection was performed using an Enhanced Chemiluminescence Detection kit according to the manufacturer's instructions. Results are expressed as Arbitrary Units (AU) normalized with *β*-actin.

### 2.9. DDAH Activity Assay

Brain homogenates were centrifuged at 5000 xg for 60 min at 4°C and supernatants were collected for DDAH activity assay, performed by determining L-citrulline formation in 96-well microtiter plate, according to Knipp's method [40]. Results are expressed as units/mg protein + S.D. One unit of enzyme activity was defined as the amount of enzyme catalyzing the formation of one mmol L-citrulline/min at 37°C. 

### 2.10. Protein Assay

Protein content was evaluated according to the method of Lowry [35].

## 3. Statistical Analysis

One-way analysis of variance (ANOVA) followed by Bonferroni's *t*-test was performed in order to estimate significant differences among groups. Data were reported as mean values ± S.D. and differences between groups were considered to be significant at *P* < 0.005.

## 4. Results

As already reported in our previous studies, this experimental procedure allowed the induction of cerebral ischemia [41–48]; in fact a significant increase in lactate levels was found in ischemic animals compared to sham-operated controls (sham-operated: 22.84 ± 3,42 nmol/mg prot, ischemic animals: 72.15 ± 2.89 nmole/mg prot. Each value represents the mean ± SD of 10 animals; *P* < 0.001).

**Figure 1:**
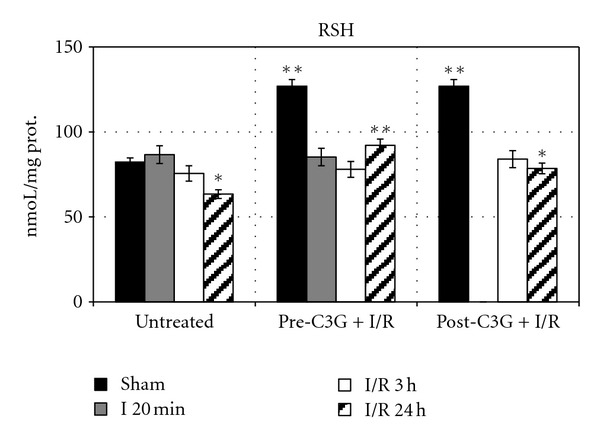
Effect of pre- and posttreatment with C3G on RSH levels in rat brain during ischemia/reperfusion injury. Each value represents the mean of 10 samples in triplicate with standard deviations represented by vertical bars. *P < 0.005 with respect to sham, untreated rats; **P < 0.001 with respect to untreated rats, same reperfusion time.

Figure 1 reports RSH levels in brain tissues of rats submitted to our experimental conditions of partial cerebral ischemia and subsequent reperfusion, both in saline-treated animals and in C3G pre- or posttreated rats. No significant change was observed in saline-treated rats after 20 min ischemia or after 3 h postischemic reperfusion respect to sham-operated animals; after 24 h postischemic reperfusion, brain RSH levels underwent to a significant 23% reduction with respect to sham-operated animals. The same figure also shows results obtained in C3G-treated animals; in sham operated animals, C3G administration induces a significant increase in RSH levels with respect to untreated rats. However, C3G treatment was not able to induce significant modifications in RSH levels in ischemic rats or after 3 h postischemic reperfusion. Significant differences in brain RSH levels are evident after prolonged postischemic reperfusion; in fact, after 24 h postischemic reperfusion, C3G-pretreated rats showed higher RSH levels with respect to untreated animals; however C3G resulted less efficient in maintaining RSH levels when administered after ischemic event (posttreatment). 

**Figure 2:**
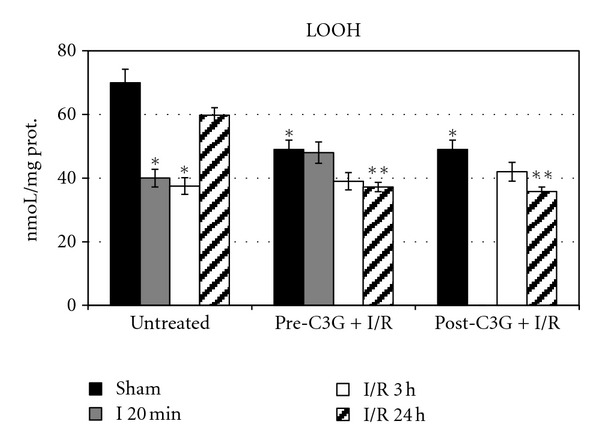
Effect of pre- and posttreatment with C3G on LOOH levels in rat brain during ischemia/reperfusion injury. Each value represents the mean of 10 samples, in triplicate, with standard deviations represented by vertical bars. *P < 0.001 with respect to sham, untreated rats; **P < 0.001 with respect to untreated rats, same reperfusion time.

Figure 2 reports LOOH levels; in saline-treated animals a significant decrease of such oxidative stress marker is evident after 20 min ischemia and after 3 h postischemic reperfusion. These results can be explained considering the need of oxygen for LOOH formation. Following 24 h postischemic reperfusion LOOH levels are similar to those observed in sham-operated rats. C3G treatment significantly reduces LOOH levels in sham-operated animals; however, after 20 min ischemia or after 3 h postischemic reperfusion, neither pretreatment nor posttreatment with C3G induced significant differences with respect to untreated rats. By contrast after 24 h postischemic reperfusion, C3G efficiently contrasted lipid peroxidation; in fact, LOOH levels resulted significantly decreased in C3G-treated animals when compared to untreated rats. 

**Figure 3:**
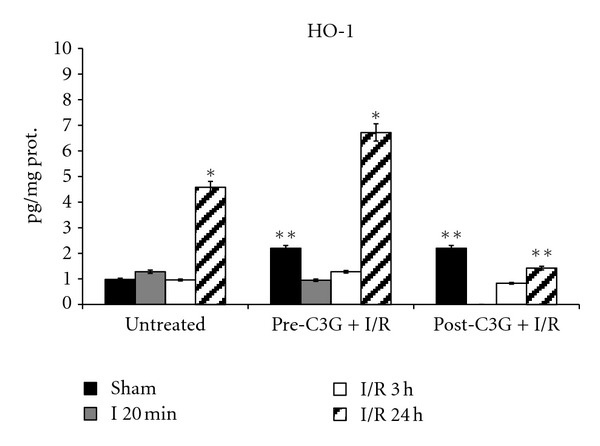
Effect of pre- and posttreatment with C3G on HO-1 protein content; *P < 0.001 versus sham; **P < 0.001 with respect to untreated rats.

Results regarding HO-1 content are reported in Figure 3. In untreated animals a significant increase in HO-1 is evident after 24 h postischemic reperfusion. C3G treatment caused a significant increase in HO-1 levels in sham-operated rats, whilst no significant difference was observed between untreated and C3G-treated animals, neither after 20 min ischemia nor after 3 h postischemic reperfusion. The HO-1 inducing effect of C3G is evident in brains of rats pretreated with C3G and submitted to 24 h postischemic reperfusion; in fact, as shown in Figure 3, when C3G is injected before ischemic insult, it increased HO-1 expression following 24 h reperfusion. This effect was not evident when C3G was administered after the ischemic injury. 

**Figure 4:**
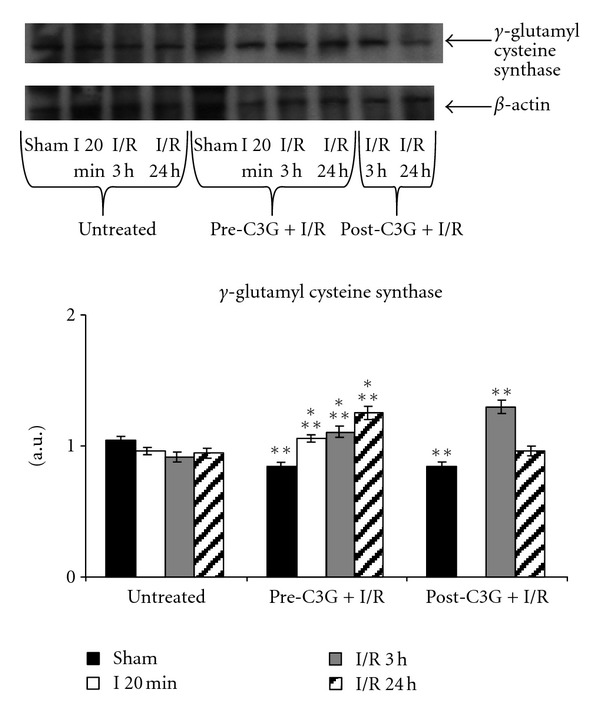
Effect of pre- and posttreatment with C3G on *γ*-GCS expression; densitometric analysis was performed after normalization with *β*-actin (S.D. is represented by vertical bars). Blots shown are representative of Western blot analysis from 4 separate experiments; *P < 0.001 versus sham-operated animals; **P < 0.005 with respect to untreated rats, same time of reperfusion.

No significant change in *γ*-GCS was observed in the brain of saline-treated rats submitted to our experimental conditions of cerebral ischemia and reperfusion (Figure 4). The same figure also shows the effect of C3G treatment: in sham operated C3G-injected animals a significant decrease in *γ*-GCS expression was observed with respect to untreated animals; however the same pretreatment resulted in a gradual and significant increase following ischemia and subsequent reperfusion particularly. The posttreatment with C3G induced a significant increase in enzyme expression after 3 h of reperfusion; following 24 h postischemic reperfusion the enzyme expression was similar to that observed in saline-treated animals at the same reperfusion time. 

**Figure 5:**
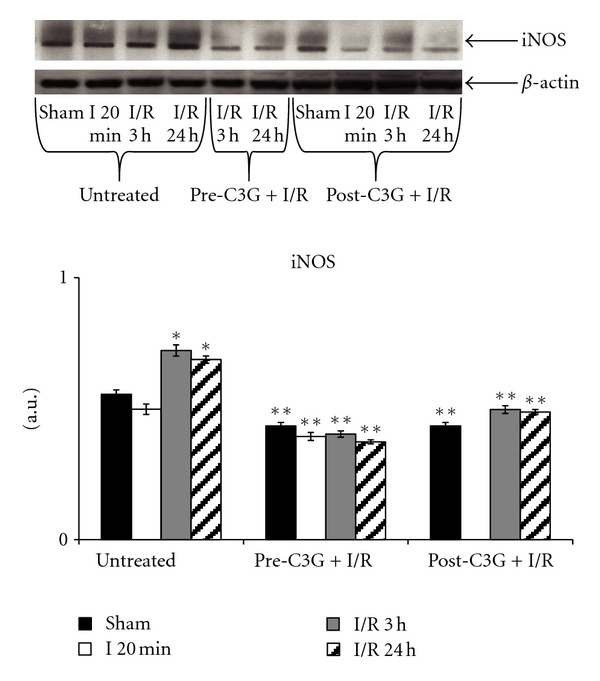
iNOS in whole brain homogenate after ischemia-reperfusion—effect of pre- and posttreatment with C3G. Protein levels were visualized by immunoblotting with antibody against iNOS. Densitometric analysis was performed after normalization with *β*-actin (S.D. is represented by vertical bars). Blots shown are representative of western blot analysis from 4 separate experiments. *P < 0.001 versus sham, **P < 0.0001 with respect to untreated rats, same time of reperfusion.

Results regarding iNOS expression in brains of rats underwent our experimental conditions of partial and transient cerebral ischemia are reported in Figure 5; iNOS is expressed in untreated, both sham-operated and ischemic animals. After 3 h and 24 h postischemic reperfusion a significant increase in iNOS expression was observed. The same figure also shows results concerning the effects of C3G treatment. As it can be seen, C3G pretreatment caused a significant reduction of iNOS expression which is evident both in sham-operated and in ischemic or postischemic reperfused rats. Similar results were obtained when C3G was injected after ischemic insult. 

**Figure 6:**
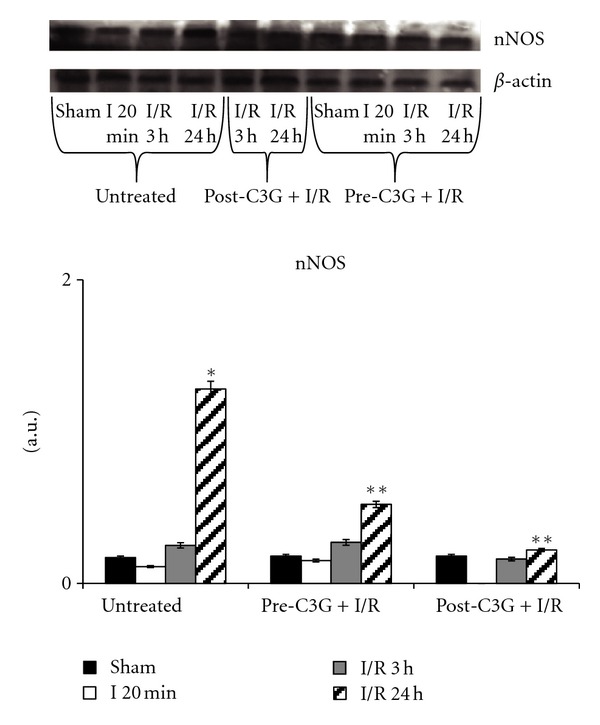
nNOS in whole brain homogenate after ischemia-reperfusion—effect of pre- and posttreatment with C3G. Protein levels were visualized by immunoblotting with antibody against nNOS. Densitometric analysis was performed after normalization with *β*-actin (S.D. is represented by vertical bars). Blots shown are representative of western blot analysis from 4 separate experiments. *P < 0.0001 versus sham-operated animals, **P < 0.001 versus untreated rats, same time of reperfusion.

The expression of the neuronal isoform of nitric oxide synthase is reported in Figure 6. In brains of untreated animals the expression of this protein is significantly augmented following 24 h reoxygenation compared to sham-operated rats. The same figure also reports the effect of C3G treatment on the expression of nNOS: no significant difference was observed between saline- and C3G-treated animals either after 20 min ischemia or after 3 h reperfusion. More relevant changes in nNOS expression were observed after 24 h postischemic reperfusion, when both pre- and posttreatment with C3G significantly decreased nNOS expression. 

**Figure 7:**
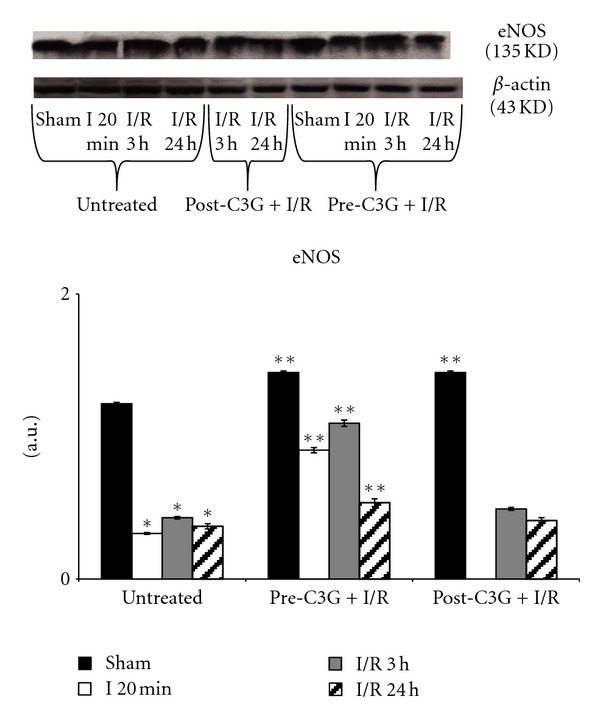
Effect of pre- and posttreatment with C3G on eNOS expression. Densitometric analysis was performed after normalization with *β*-actin (S.D. is represented by vertical bars). Blots shown are representative of Western blot analysis from 4 separate experiments; *P < 0.001 versus sham; **P < 0.001 with respect to untreated rats.

The pattern of eNOS expression, reported in Figure 7, showed a significant decrease in untreated animals following 20 min ischemia and after 3 or 24 h reperfusion. C3G treatment induced a significant increase in eNOS expression in sham-operated animals; however, only the pretreatment was able to maintain high levels of expression of eNOS after ischemia and 3 or 24 h reperfusion; in fact, C3G posttreated rats showed eNOS levels similar to those observed in untreated animals. 

**Figure 8:**
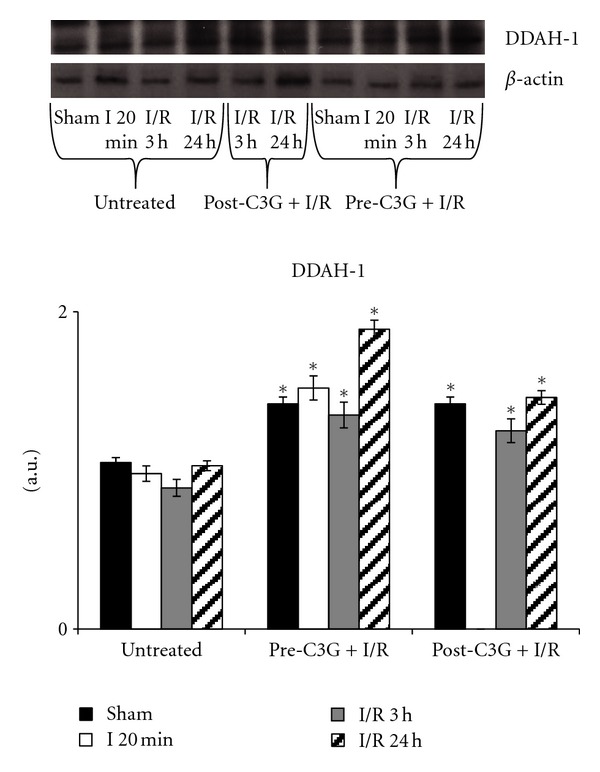
Effect of pre- and posttreatment with C3G on DDAH-1 expression. Densitometric analysis was performed after normalization with *β*-actin (S.D. is represented by vertical bars). Blots shown are representative of Western blot analysis from 4 separate experiments; *P < 0.005 with respect to untreated group.

Western blot analysis of cerebral contents in DDAH-1 evidenced that this protein was poorly modified under our experimental conditions of cerebral postischemic reperfusion. In fact, as shown in Figure 8, no significant change in DDAH-1 levels was observed in saline-treated animals between sham-operated and ischemic, 3 h or 24 h postischemic reperfused rats. C3G both pre- and posttreatment induced a significant increase in DDAH-1 expression with respect to saline-treated rats. Highest levels of DDAH-1 were observed in brains of rats C3G-pretreated and reperfused for 24 hs.

**Figure 9:**
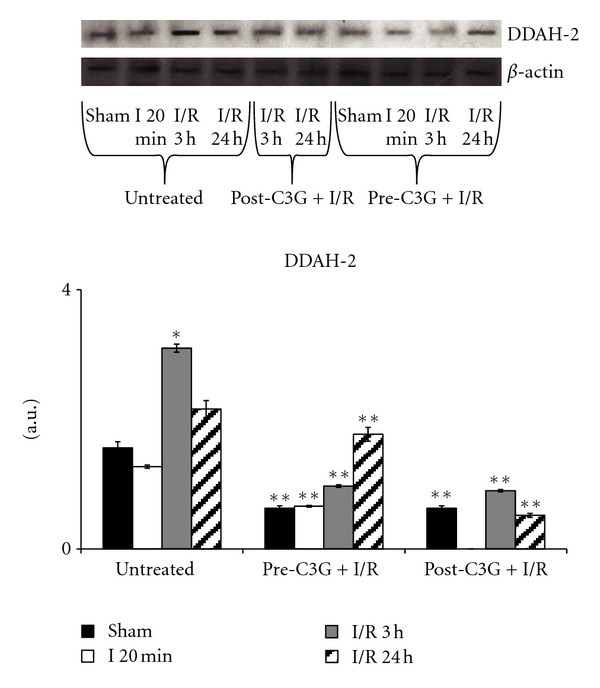
Effect of pre- and posttreatment with C3G on DDAH-2 expression. Densitometric analysis was performed after normalization with *β*-actin (S.D. is represented by vertical bars). Blots shown are representative of western blot analysis from 4 separate experiments; *P < 0.001 versus untreated, sham operated rats; **P < 0.001 versus untreated group.

Very relevant resulted the changes observed in DDAH-2 expression and reported in Figure 9. In untreated rats, DDAH-2 expression significantly increased after 3 h reperfusion and then trended to reduce again to control values. C3G treatment caused a significant DDAH-2 reduction in sham-operated animals and this trend was also kept under ischemia/reperfusion conditions, both following pretreatment and post-injection with C3G. 

**Figure 10:**
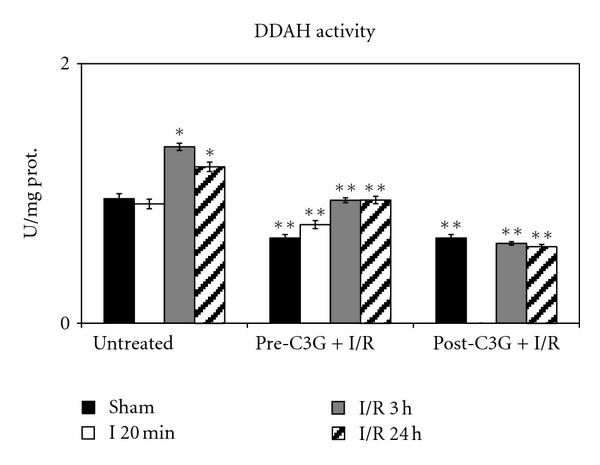
Effect of pre- and posttreatment with C3G on DDAH activity. Values are means of 3 determinations/sample (10 samples/group), with standard deviations represented by vertical bars. *P < 0.001 versus sham, untreated rats; **P < 0.001 with respect to untreated group, same reperfusion time.

Results regarding the determination of DDAH activity are reported in Figure 10: the pattern resembles DDAH-2 expression, both in untreated and in C3G-treated animals. 

**Figure 11:**
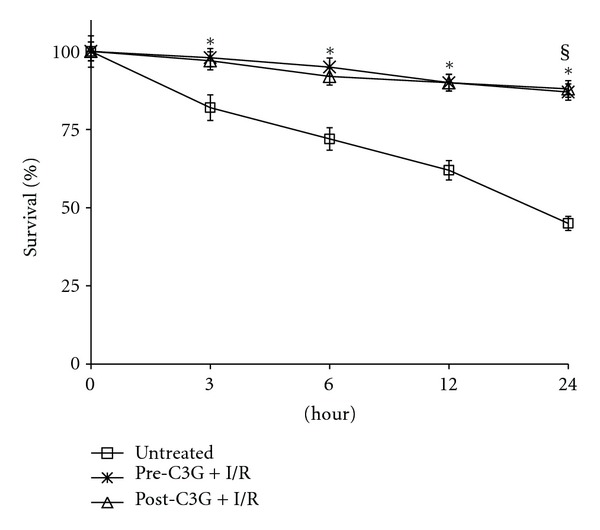
Effect of pre- and posttreatment with C3G on survival percentages of rats after 20 min cerebral ischemia. Values are means of 30 animals/group with standard deviations represented by vertical bars.** §** In sham operated rats survival was 100% (both untreated and C3G-treated); *P < 0.001 with respect to untreated group, same reperfusion time.

The administration of C3G significantly increased the survival rate of rats, both if administered before and after ischemic injury (Figure 11). In sham-operated rats, both untreated and C3G-treated the survival rate was 100% (Figure 11).

## 5. Discussion

Stroke represents one of the major causes of death or invalidity in developed countries. In most cases, stroke results from the obstruction of blood flow in a major cerebral vessel. Understanding biochemical mechanisms involved in brain damage subsequent to ischemic injury is crucial for developing new therapies. After ischemic insult, neuronal cell death proceeds through a mixture of mechanisms including excitoxicity, apoptosis, inflammation and oxidative stress [15]. The occurrence of this cascade of events was also demonstrated under *in vivo* experimental conditions of partial and transient cerebral ischemia in rats and was indirectly confirmed by protective effects observed following treatment with drugs acting with different molecular mechanisms [41–46]. Over recent years, neuroscientists have acquired a considerable body of evidence to support the fact that the mammalian brain can adapt to injurious insults such cerebral ischemia, thus increasing the chances of survival [47, 48]. So, sublethal ischemic insults may protect tissues from subsequent insults. This phenomenon is known as *preconditioning* or i*schemic tolerance* (also defined as a short, sublethal ischemic episode that activates endogenous mechanisms able to protect organs or tissues from further longer and more severe episodes of ischemia); while it would be dangerous and impractical to precondition at-risk patients with ischemia, elucidation of the endogenous cell survival pathways involved in ischemic tolerance has many clinical implications and may lead to new therapeutic strategies. HO-1 (also known as stress protein HSP32) can be over-expressed in many tissues following stressful stimuli including hypoxia, hyperoxia, ischemia-reperfusion, and a wide range of conditions characterized by alteration of the cellular redox state [22, 28, 49–55]. HO-1 expression might represent an important protective endogenous mechanism; in this regard, induction of this enzyme has shown beneficial effects in several pathological conditions [56, 57]. In this context, pharmacologic modulation of HO-1 system may represent an effective strategy to intervene in several pathologic conditions but it is important to induce HO-1 expression without causing cell damages. Recently the ability of several natural antioxidants to induce HO-1 has been reported [27, 58–62]. The anthocyanin are part of the widespread group of plant constituents, collectively known as flavonoids. Cyanidin-3-O-*β*-glucoside, also known as kuromanin, is probably the best known and most investigated cyanidin-glycoside. There are several reports mentioning beneficial effects of C3G, such as prevention of LDL oxidation, cardiovascular diseases, inflammation and obesity, vascular failure, and myocardium damage, besides the well-known free radical-scavenging activity [32, 63–71]. Results obtained in the present study confirmed antioxidant properties of C3G and also suggested that it was not merely attributable to its antioxidant activity. 

Interestingly, the effect of C3G treatment on *γ*-GCS expression seemed to be related to cellular needs; in fact, the significant reduction in *γ*-GCS expression observed in C3G-treated sham-operated animals suggested that under physiological condition the antioxidant activity of C3G made cells adequately protected from oxidant; however, the same treatment was able to induce significant increases in *γ*-GCS in animals underwent to ischemic/reperfusion damage thus enhancing glutathione (GSH) levels and their defences against oxidative stress. 

Our previous studies, carried out on the same experimental model of partial and transient cerebral ischemia, indicated that the environment of injured neurons seemed to determine the ability of axons to regenerate after injury [47, 72]. To this regard, endogenous mediators affecting vasculature such as endothelium-derived NO, inducing smooth muscle relaxation and then vasodilatation of brain vessels, might improve tissue perfusion, attenuating the ischemic insult and promoting functional recovery of the infarcted brain area. Consequently, upregulation of eNOS may serve a protective role by facilitating the maintenance of cerebral blood flow and promoting revascularisation after an ischemic insult. Here reported data demonstrated that C3G treatment was able to induce e-NOS expression both in sham-operated animals and in ischemic/reperfused rats (although pretreatment had more efficient results). In addition it has to be noted that C3G treatment evoked other very important effects on NO pathway. Large amounts of NO produced by nNOS and iNOS contribute to metabolic deterioration and adversely affect the ischemic brain. Results obtained in the present study demonstrated the ability of C3G administration to significantly decrease iNOS and nNOS expressions. One of the more relevant effects of C3G treatment in postischemic reperfusion brain damage was probably due just to its ability to discriminate between endothelial or inducible/neuronal NOS isoforms. Numerous reports in the literature indicated that endogenously produced inhibitors of NOS, particularly ADMA, regulate NO generation [14, 73, 74]; ADMA levels, in turn, are highly regulated by DDAH enzymes, which are responsible for ADMA metabolism. Thus, activation or hyperexpression of DDAHs, resulting in enhanced catabolism of the endogenous NOS inhibitor ADMA, lead to increased amounts of NO. Conversely, specific inhibitors of DDAH activity or expression induce an accumulation of the inhibitor and then smaller amounts of NO.

Results obtained in the present study evidenced no significant change in DDAH-1 expression in untreated, ischemic/reperfused rats. In the same experimental group the observed changes in enzymatic activity appeared to be mainly due to DDAH-2isoform. In fact enzyme activity showed the same trend of DDAH-2 expression, both in untreated and in C3G-injected rats. In this regard, particularly interesting was the significant decrease observed both in DDAH-2 expression and DDAH activity of C3G-treated rats. Given the pleiotropic effect of NO during cerebral ischemia, DDAH has the potential to regulate all the effects of NO through modulation of ADMA levels. Another equally relevant result was that cyanidin was able to produce its effects even when administered after ischemic insult, not only when given before ischemia. 

The ability of C3G to inhibit DDAH activity and expression, along with the capacity to selectively affect different NOS isoforms strongly suggested an important therapeutic role of this anthocyanin also confirmed by the significant increase in survival rate, observed both in C3G -pretreated and post-treated rats. The multiplicity of effects observed in C3G-treated rats indicated a complex mode of action of the natural molecule involving several pathways. All these features, according to reports of Min et al. [75], suggested that it might be very useful both in prevention and therapy of postischemic reperfusion brain damage.
